# Supplementary material for: Development and initial validation of the Psychological Need Frustration Scale for Physical Activity
Source: PeerJ. 2020 May 28;8:e9210. doi: 10.7717/peerj.9210 (PMC7265887; doi:10.7717/peerj.9210)
Supplement: Supplemental Information 4 [file peerj-08-9210-s004.docx]

A codebook of converted numbers

| Variables | Converted numbers |
| --- | --- |
| Sex | 1.00 = Male ; 2.00 = Female |
|  |  |
| Psychological Need Frustration Scale for Physical Activity | 1. = Strongly disagree   7.0 = Strongly agree |
