# Supplementary material for: Development and initial validation of the Psychological Need Frustration Scale for Physical Activity
Source: PeerJ. 2020 May 28;8:e9210. doi: 10.7717/peerj.9210 (PMC7265887; doi:10.7717/peerj.9210)
Supplement: Supplemental Information 5 [file peerj-08-9210-s005.docx]

Psychological Need Frustration Scale for Physical Activity

|  | | Strongly disagree ▪▪▪▪▪▪▪▪▪▪▪▪ Strongly agree | | | | | | |
| --- | --- | --- | --- | --- | --- | --- | --- | --- |
|  | (Autonomy) When engaging in physical activity, at times you feel: |  |  |  |  |  |  |  |
| 1 | Restricted from making choice. | 1 | 2 | 3 | 4 | 5 | 6 | 7 |
| 2 | Forced to follow decisions made for you. | 1 | 2 | 3 | 4 | 5 | 6 | 7 |
| 3 | Other people make their demand without providing rationale. | 1 | 2 | 3 | 4 | 5 | 6 | 7 |
| 4 | Other people use excessive personal control. | 1 | 2 | 3 | 4 | 5 | 6 | 7 |
| 5 | Forced to do things that you don’t want to. | 1 | 2 | 3 | 4 | 5 | 6 | 7 |
|  | (Competence) When engaging in physical activity, at times: |  |  |  |  |  |  |  |
| 1 | You are made to feel powerless in some situations. | 1 | 2 | 3 | 4 | 5 | 6 | 7 |
| 2 | You feel incompetent because of things you are told. | 1 | 2 | 3 | 4 | 5 | 6 | 7 |
| 3 | You doubt if you can achieve improvement because of comments you receive. | 1 | 2 | 3 | 4 | 5 | 6 | 7 |
| 4 | You doubt your ability to overcome challenges because of comments you receive. | 1 | 2 | 3 | 4 | 5 | 6 | 7 |
|  | (Relatedness) When engaging in physical activity, at times you feel: |  |  |  |  |  |  |  |
| 1 | You are rejected by those around you. | 1 | 2 | 3 | 4 | 5 | 6 | 7 |
| 2 | Other people overlook you on purpose. | 1 | 2 | 3 | 4 | 5 | 6 | 7 |
| 3 | Other people say bad words about you. | 1 | 2 | 3 | 4 | 5 | 6 | 7 |
| 4 | Other people do not listen to you. | 1 | 2 | 3 | 4 | 5 | 6 | 7 |
| 5 | Other people are reluctant to offer help. | 1 | 2 | 3 | 4 | 5 | 6 | 7 |

身体活动中的基本心理需要挫败感量表

|  | | 非常不同意 ▪▪▪▪▪▪▪▪▪▪▪▪ 非常同意 | | | | | | |
| --- | --- | --- | --- | --- | --- | --- | --- | --- |
|  | (自主感) 在你進行身體活動時，有時候你感覺： |  |  |  |  |  |  |  |
| 1 | 別人限制你做出選擇。 | 1 | 2 | 3 | 4 | 5 | 6 | 7 |
| 2 | 要被迫服從別人為你作出的身體活動決定。 | 1 | 2 | 3 | 4 | 5 | 6 | 7 |
| 3 | 別人給你提出要求卻不說明理由。 | 1 | 2 | 3 | 4 | 5 | 6 | 7 |
| 4 | 別人對你進行過多的控制。 | 1 | 2 | 3 | 4 | 5 | 6 | 7 |
| 5 | 你被迫做你不想做的事。 | 1 | 2 | 3 | 4 | 5 | 6 | 7 |
|  | (能力感) 在你進行身體活動時，有時候： |  |  |  |  |  |  |  |
| 1 | 有一些情形讓你感到力不從心。 | 1 | 2 | 3 | 4 | 5 | 6 | 7 |
| 2 | 別人告訴你一些事，讓你感到自己能力不足。 | 1 | 2 | 3 | 4 | 5 | 6 | 7 |
| 3 | 別人對你的一些評價，使你懷疑自己能否取得進步。 | 1 | 2 | 3 | 4 | 5 | 6 | 7 |
| 4 | 別人對你的一些評價，使你懷疑自己克服困難的能力。 | 1 | 2 | 3 | 4 | 5 | 6 | 7 |
|  | (關聯感) 在你進行身體活動時，有時候你感覺： |  |  |  |  |  |  |  |
| 1 | 別人排斥你。 | 1 | 2 | 3 | 4 | 5 | 6 | 7 |
| 2 | 別人故意忽視你。 | 1 | 2 | 3 | 4 | 5 | 6 | 7 |
| 3 | 別人說你的壞話。 | 1 | 2 | 3 | 4 | 5 | 6 | 7 |
| 4 | 別人不傾聽你的想法。 | 1 | 2 | 3 | 4 | 5 | 6 | 7 |
| 5 | 別人不願幫助你。 | 1 | 2 | 3 | 4 | 5 | 6 | 7 |
